# Supplementary material for: A one-step foraminoplasty via a large trephine in percutaneous endoscopic transforaminal discectomy for the treatment of lumbar disc herniation
Source: PLoS One. 2022 May 24;17(5):e0268564. doi: 10.1371/journal.pone.0268564 (PMC9128989; doi:10.1371/journal.pone.0268564)
Supplement: Attachment — Submitted filename: Response to Reviewers.docx [file pone.0268564.s003.docx]

Dr. **Bo Wang**

Department of Spine Surgery, Beijing Jishuitan Hospital, Beijing 100035, P.R. China

Tel: +86-10-58517207

Email: drwangbo@pku.edu.cn

Dear Editor,

Thank you for your letter of comments concerning our manuscript entitled ‘A one-step foraminoplasty via a large trephine in percutaneous endoscopic transforaminal discectomy for the treatment of lumbar disc herniation’. We are grateful to the editor and the reviewers for carefully considering our manuscript and the valuable comments. We have studied the comments carefully and have made correction which we hope meet with the approval.

Thank you again for your kind attention to our manuscript. We have resubmitted the revised manuscript to the journal. We deeply appreciate your reconsideration of our manuscript, and look forward to your positive response.

Kind regards,

Bo Wang

***Reply to the reviewer’s comments***

1. Perhaps, it would be very interesting if you could offer to readers a side-by-side illustrated comparison between both the treatment procedures.

RE: Thanks for the reviewer’s positive comments. We offered an illustration of the new procedures and it would be more interesting to offer the illustration of the traditional procedures side-by-side. However, the traditional step-by-step intervertebral foramen forming technology has been used for many years as a mature technical, which is well known to spinal surgeons. In view of this, considering the costs of time, we are sorry that we didn’t offer the compared illustration.

2. Please replace the following:
Line 98 - surgical area; should be replaced by surgical site;
Line 333 - with central dis herniation should be replaced by central disc herniation
RE: Thanks for the detailed suggestion. We are so sorry for the errors and the word mentioned above has been replaced.

***Reply to the Journal Requirements:***

When submitting your revision, we need you to address these additional requirements.

1. Please ensure that your manuscript meets PLOS ONE's style requirements, including those for file naming. The PLOS ONE style templates can be found at

<https://journals.plos.org/plosone/s/file?id=wjVg/PLOSOne_formatting_sample_main_body.pdf>  and

<https://journals.plos.org/plosone/s/file?id=ba62/PLOSOne_formatting_sample_title_authors_affiliations.pdf>

RE: Thank you. The revised manuscript has been checked to meet the PLOS ONE’s style requirements.

2. Please amend your current ethics statement to address the following concerns:

a) Did participants provide their written or verbal informed consent to participate in this study?

b) If consent was verbal, please explain i) why written consent was not obtained, ii) how you documented participant consent, and iii) whether the ethics committees/IRB approved this consent procedure.

RE: The written informed consent was obtained from the participations and the ethics statement has been amended.

3. Please include your full ethics statement in the ‘Methods’ section of your manuscript file. In your statement, please include the full name of the IRB or ethics committee who approved or waived your study, as well as whether or not you obtained informed written or verbal consent. If consent was waived for your study, please include this information in your statement as well.

RE: The full ethics statement has been added in the ‘Methods’ section in the revised manuscript.

4. Please ensure that you refer to Figure 5 in your text as, if accepted, production will need this reference to link the reader to the figure.

RE: Sorry for the mistake. The ‘Figure 2’ in the ‘3.3 Clinical outcome evaluation’ part has been corrected to ‘Figure 5’.

5. In your Data Availability statement, you have not specified where the minimal data set underlying the results described in your manuscript can be found. PLOS defines a study's minimal data set as the underlying data used to reach the conclusions drawn in the manuscript and any additional data required to replicate the reported study findings in their entirety. All PLOS journals require that the minimal data set be made fully available. For more information about our data policy, please see http://journals.plos.org/plosone/s/data-availability.

Upon re-submitting your revised manuscript, please upload your study’s minimal underlying data set as either Supporting Information files or to a stable, public repository and include the relevant URLs, DOIs, or accession numbers within your revised cover letter. For a list of acceptable repositories, please see http://journals.plos.org/plosone/s/data-availability#loc-recommended-repositories. Any potentially identifying patient information must be fully anonymized.

Important: If there are ethical or legal restrictions to sharing your data publicly, please explain these restrictions in detail. Please see our guidelines for more information on what we consider unacceptable restrictions to publicly sharing data: http://journals.plos.org/plosone/s/data-availability#loc-unacceptable-data-access-restrictions. Note that it is not acceptable for the authors to be the sole named individuals responsible for ensuring data access.

We will update your Data Availability statement to reflect the information you provide in your cover letter.

RE: Thank you. The Data Availability statement has been updated and the study’s minimal underlying data set was uploaded as the Supporting Information.

6. Please review your reference list to ensure that it is complete and correct. If you have cited papers that have been retracted, please include the rationale for doing so in the manuscript text, or remove these references and replace them with relevant current references. Any changes to the reference list should be mentioned in the rebuttal letter that accompanies your revised manuscript. If you need to cite a retracted article, indicate the article’s retracted status in the References list and also include a citation and full reference for the retraction notice.
RE: Thank you. The reference list has been reviewed.
